# Sex-specific effects of aging on humoral immune responses to repeated influenza vaccination in older adults

**DOI:** 10.1038/s41541-021-00412-6

**Published:** 2021-12-09

**Authors:** Janna R. Shapiro, Huifen Li, Rosemary Morgan, Yiyin Chen, Helen Kuo, Xiaoxuan Ning, Patrick Shea, Cunjin Wu, Katherine Merport, Rayna Saldanha, Suifeng Liu, Engle Abrams, Yan Chen, Denise C. Kelly, Eileen Sheridan-Malone, Lan Wang, Scott L. Zeger, Sabra L. Klein, Sean X. Leng

**Affiliations:** 1 Department of International Health, Johns Hopkins Bloomberg School of Public Health, Baltimore, MD USA; 2 Division of Geriatric Medicine and Gerontology, Department of Medicine, Johns Hopkins University School of Medicine, Baltimore, MD USA; 3 Guangdong Geriatrics Institute, Guangdong Provincial People’s Hospital, Guangdong Academy of Medical Sciences, Guangzhou, Guangdong China; 4 Department of Geriatrics, Xijing Hospital, The Fourth Military Medical University, Xi’an, Shaanxi China; 5 W. Harry Feinstone Department of Molecular Microbiology and Immunology, Johns Hopkins Bloomberg School of Public Health, Baltimore, MD USA; 6 Department of Geriatrics, The Second Hospital of Tianjin Medical University, Tianjin, Hebei China; 7 Zanvyl Krieger School of Arts and Science, Johns Hopkins University, Baltimore, MD USA; 8 Zhongshan Hospital, Xiamen University, Xiamen, Fujian China; 9 Department of Geriatrics, The First Affiliated Hospital of Chongqing Medical University, Chongqing, Sichuan China; 10 Department of Geriatrics, The First Affiliated Hospital, Zhejiang University School of Medicine, Hangzhou, Zhejiang, China; 11 Department of Biostatistics, Johns Hopkins Bloomberg School of Public Health, Baltimore, MD USA

**Keywords:** Antibodies, Inactivated vaccines

## Abstract

Older adults (≥65 years of age) bear a significant burden of severe disease and mortality associated with influenza, despite relatively high annual vaccination coverage and substantial pre-existing immunity to influenza. To test the hypothesis that host factors, including age and sex, play a role in determining the effect of repeated vaccination and levels of pre-existing humoral immunity to influenza, we evaluated pre- and post-vaccination strain-specific hemagglutination inhibition (HAI) titers in adults over 75 years of age who received a high-dose influenza vaccine in at least four out of six influenza seasons. Pre-vaccination titers, rather than host factors and repeated vaccination were significantly associated with post-vaccination HAI titer outcomes, and displayed an age-by-sex interaction. Pre-vaccination titers to H1N1 remained constant with age. Titers to H3N2 and influenza B viruses decreased substantially with age in males, whereas titers in females remained constant with age. Our findings highlight the importance of pre-existing immunity in this highly vaccinated older adult population and suggest that older males are particularly vulnerable to reduced pre-existing humoral immunity to influenza.

## Introduction

Seasonal influenza is an important public health burden in older adults (people ≥ 65 years of age), particularly the oldest and frail subset^1–3^. In the United States (U.S.), there are an estimated 4 million incident cases per year in older adults, accounting for 90% of deaths associated with influenza^4,5^. The U.S. Centers for Disease Control and Prevention (CDC) recommends annual influenza vaccination for prevention of influenza infection and complications in people 6 months and older^6^. The high-dose inactivated influenza vaccine (HD-IIV) is available to older adults and has demonstrated superior efficacy over the standard-dose vaccine in older age groups^6,7^. Seasonal influenza vaccination coverage is relatively high in older adults, with >60% of older Americans being vaccinated annually, compared to <40% vaccination coverage in the 18–49 age group^8^.

Age-related immunosenescence, defined by a decline in cellular and humoral immune function combined with a chronic low-grade inflammatory phenotype (CLIP), or inflammaging^9–11^, is believed to be the primary reason for the reduced effectiveness of influenza vaccines observed in older adults^12–14^. Repeated annual vaccination may also have a negative effect on vaccine-induced humoral immune responses as well as vaccine effectiveness (VE). For example, a recent observational test-negative study using ten years of vaccination history found that in older adults, VE decreases with increasing numbers of previous vaccinations but that vaccination continues to offer some level of protection^15^. Another study over eight seasons in the general adult population found that VE to H3N2, but not influenza B virus, is reduced among individuals with frequent vaccination history compared to those without prior vaccination^16^. Age-specific effects have also been observed in this context, with a reduction in influenza vaccine immunogenicity observed with repeat vaccination in teenagers, but not adults^17^. In addition, a meta-analysis found heterogeneous effects of repeated vaccination overall and that when negative effects are observed, they are most pronounced for H3N2^18^. In contrast, a recent systematic review and meta-analysis concluded that the available evidence did not support a reduction in VE with consecutive repeat vaccination, but that certainty in the evidence was low^19^. Case–control studies in both Australia and Spain found beneficial effects of repeated annual vaccination on VE in older adults^20,21^, and an observational population-based study in Sweden found no differences in VE between those who had been vaccinated in the current season only and those who had been vaccinated in both the current and previous seasons^22^. Based on the conflicting evidence, multi-season clinical studies to address the effects of aging and repeated vaccination have been recommended^18^.

Mechanistically, pre-existing immunity generated to various influenza virus exposures over time can have an important impact on the outcome of vaccination. According to the immune imprinting theory, the memory response established by an individual’s first influenza exposure has a lifelong effect on subsequent immune responses to infection or vaccination^23^. Broad pre-existing immunity is thought to have negative consequences, as pre-existing antibodies can suppress the response to novel influenza virus strains by reducing the amount of available antigen or epitope masking^24,25^. A theoretical benefit of HD-IIV is that pre-existing antibodies cannot sequester the increased amount of antigen delivered, and, thus, more antigen is available to activate memory B cells and elicit a protective response^26–28^. To our knowledge, however, the impact of pre-existing immunity in the context of HD-IIV has not been adequately characterized.

In addition to age, other host factors including sex, frailty, and body mass index (BMI) can impact vaccine responses in older adults. Females have been found to mount greater antibody responses to HD-IIV than males^29^. The immunological differences between males and females in immune responses are largely attributed to sex hormones and sex chromosomes^30–32^. Inflammaging also contributes significantly to age- and sex-related differences in immune responses and vulnerability to infections, as discussed in two recent reviews in the context of SARS-CoV-2 infection and ongoing pandemic^31,33^. The relationship between frailty and influenza vaccine responses is debated in the literature, with one study reporting frailty having a negative effect^34^, others reporting no effect^35–39^, and others still reporting a positive effect^40^. Finally, in older adults, obesity, as measured by BMI, is significantly associated with decreased hemagglutination inhibition (HAI) titers and percentage of switched memory B cells^41^. Whether host related factors, including sex and age, explain variation in pre-existing immunity following repeated vaccination has not been reported. We hypothesize that the variation across studies in estimates of the effects of pre-existing immunity and repeated annual vaccination is partly caused by failure to adequately account for heterogeneity and interactions among host factors that likely differ across studies. To address this knowledge gap, we used a longitudinal cohort of older adults over 75 years of age who had received high-dose, trivalent inactivated influenza vaccine (HD-IIV3) in at least four out of six influenza seasons to estimate the impact of repeated vaccination on the antibody response to HD-IIV3 and its dependence on the intersection of age, sex, frailty, BMI, and pre-existing immunity.

## Results

### Study participants and annual influenza immunization with HD-IIV3

**Fig. 1:**
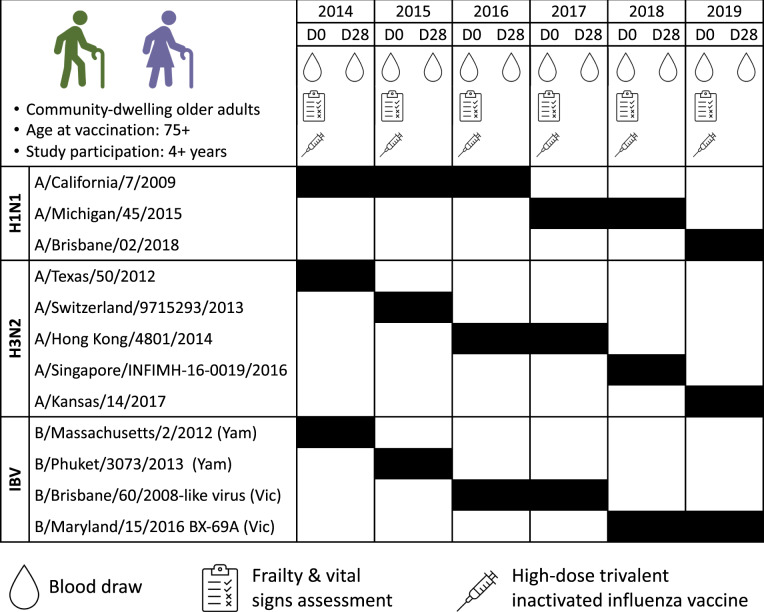
Study design. Study procedures and the three strains included in each seasonal HD-IIV3 are shown. Serum from blood draws was used to evaluate pre- and post-vaccination strain-specific hemagglutination antibody inhibition (HAI) titers, and frailty was assessed using the Frailty Phenotype. Images were created with BioRender.com.

Over the six influenza seasons from 2014–2015 to 2019–2020, 90 individuals participated in at least four study seasons and 433 doses of HD-IIV3 were administered. The strains included in each vaccine and the study protocol are described in Fig. 1. Table 1 shows demographic and clinical characteristics of the study participants. There were slightly more females (55.6%), and yearly study enrollment increased over time. There were missing data in our study (i.e., individuals who did not participate in all six influenza seasons), but this did not substantially depart from the missing at random assumption, so multi-level models were used to account for this missingness. Baseline characteristics, measured during the first year of participation, were similar between males and females. The median age at study enrollment was 80, and >50% of participants were classified as pre-frail as per the Fried Frailty Phenotype^42^. Trends in the change of frailty status since baseline were calculated as the difference between the first and last years of participation and differed by sex. A greater proportion of males improved in frailty status, whereas more females either did not change or progressed in frailty status. A greater proportion of males also experienced changes in BMI over the course of the study, while BMI did not change for females.

**Table 1:** Summary of study population characteristics.

	All	Male	Female
*Person-seasons—n (%)*	433	192 (44.3)	241 (55.7)
*Individuals—n (%)*	90	40 (44.4)	50 (55.6)
*Yearly participation—n (%)*			
2014	45 (50.0)	19 (47.5)	26 (52.0)
2015	68 (75.6)	28 (70.0)	40 (80.0)
2016	68 (75.6)	31 (77.5)	37 (74.0)
2017	87 (96.7)	38 (95.0)	49 (98.0)
2018	88 (97.8)	40 (100.0)	48 (96.0)
2019	77 (85.6)	36 (90.0)	41 (82.0)
*Number of seasons participated*			
4	40 (44.4)	18 (45.0)	22 (44.0)
5	27 (30.0)	12 (30.0)	15 (30.0)
6	23 (25.6)	10 (25.0)	13 (26.0)
*Birth year—median (p25-p75)*	1934 (1930–1938)	1934 (1929–1938)	1934 (1930–1938)
Range	1916–1941	1922–1941	1916–1940
*Baseline characteristics*^a^			
Age—Median (p25-p75)	80 (77–83)	80 (77–84)	80 (77–83)
Frailty—*n* (%)			
Non-frail	37 (41.1)	17 (42.5)	20 (40.0)
Pre-frail	48 (53.3)	21 (52.5)	27 (54.0)
Frail	5 (5.6)	2 (5.0)	3 (6.0)
BMI—median (p25-p75)	26.8 (24.5–30.4)	27.0 (25.3–29.6)	26.5 (23.2–30.5)
*Change from baseline*^b^			
Frailty—*n* (%)			
Improved	11 (12.2)	8 (20.0)	3 (6.0)
No change	44 (48.9)	18 (45.0)	26 (52.0)
Worsened	35 (38.9)	14 (35.0)	21 (42.0)
BMI			
Decreased	39 (43.3)	19 (47.5)	20 (40.0)
No change (+/−1)	38 (42.2)	14 (35.0)	24 (48.0)
Increased	13 (14.4)	7 (17.5)	6 (12.0)

^a^Value for first year participated.

^b^Difference between first and last year participated.

### Pre- and post-vaccination strain-specific HAI titers are high among repeatedly vaccinated older adults

Pre- and post-vaccination strain-specific HAI titer outcomes to the three HD-IIV3 vaccine strains are summarized and disaggregated by sex in Table 2. Pre- and post-vaccination outcomes stratified by influenza season are detailed in supplement tables (Supplementary Tables 1–6). As expected in this highly vaccinated elderly population, titers and seroprotection rates (defined as an HAI titer ≥40^43–45^) were high. This was particularly true for influenza B, where 98% of the participants were seroprotected prior to immunization. Post-vaccination, >94% of participants achieved seroprotection for H1N1 and H3N2, while 100% of participants were seroprotected against influenza B. Because of the lack of variability in post-vaccination seroprotection, this outcome was omitted from further analysis. Fold-rise in titers and rates of seroconversion, as defined by ≥4-fold titer increase^46^, were relatively low but were highest for H3N2. There were no sex differences in any post-vaccination outcomes. Together, these data indicate that pre-existing and post-vaccination strain-specific HAI titers remained high among older adults who were repeatedly vaccinated with HD-IIV3.

**Table 2:** Pre- and post-vaccination hemagglutination antibody inhibition (HAI) titer outcomes.

	All	Males	Females	Sex difference^a^
*Person-seasons—n (%)*	433	192 (44.3)	241 (55.7)	
*H1N1*				
Pre-vaccination—GMT (95% CI)	74.3 (66.8–82.8)	81.7 (70.7–94.4)	69.0 (59.0–80.6)	0.4451
Post-vaccination—GMT (95% CI)	192.3 (174.6–211.9)	185.9 (161.5–213.9)	197.7 (173.0–225.9)	0.8922
Pre-vaccination SPR—*n* (%)	325 (75.1)	149 (77.6)	176 (73.0)	0.4116
Post-vaccination SPR—*n* (%)	415 (95.8)	184 (95.8)	231 (95.9)	0.7837
Fold-rise (log10)—mean (95% CI)	0.413 (0.376–0.450)	0.357 (0.308–0.406)	0.457 (0.405–0.510)	0.1711
Seroconversion rate—*n* (%)	134 (30.9)	47 (24.5)	87 (36.1)	0.1932
*H3N2*				
Pre-vaccination—GMT (95% CI)	89.3 (77.0–103.5)	91.8 (73.0–115.4)	87.3 (71.9–106.0)	0.8246
Post-vaccination—GMT (95% CI)	363.1 (316.3–416.8)	365.0 (292.3–455.9)	361.5 (303.5–430.6)	0.8793
Pre-vaccination SPR—*n* (%)	314 (72.5)	141 (73.4)	173 (71.8)	0.8855
Post-vaccination SPR—*n* (%)	408 (94.2)	179 (93.2)	229 (95.0)	0.4556
Fold-rise (log10)—mean (95% CI)	0.609 (0.559–0.660)	0.600 (0.527–0.672)	0.617 (0.548–0.687)	0.8554
Seroconversion rate—*n* (%)	207 (47.8)	84 (43.8)	123 (51.0)	0.1807
*B*				
Pre-vaccination—GMT (95% CI)	262.8 (235.3–293.4)	236.3 (204.8–272.6)	286.0 (243.1–336.4)	0.2847
Post-vaccination—GMT (95% CI)	571.1 (520.2–627.1)	508.5 (445.8–580.1)	626.5 (549.7–714.0)	0.1735
Pre-vaccination SPR—*n* (%)	424 (97.9)	191 (99.5)	233 (96.7)	0.2887
Post-vaccination SPR—*n* (%)	433 (100.0)	192 (100.0)	241 (100.0)	
Fold-rise (log10)—mean (95% CI)	0.337 (0.304–0.370)	0.333 (0.288–0.378)	0.341 (0.294–0.387)	0.9482
Seroconversion rate—*n* (%)	105 (24.2)	47 (24.5)	58 (24.1)	0.8386

CI: confidence interval; Fold-rise: post-vaccination titer divided by pre-vaccination titer, transformed on the log10 scale; GMT: geometric mean titer; SPR: seroprotection rate, the proportion of individuals who achieved a titer ≥40; Seroconversion rate: the proportion of individuals who achieved a fold-rise in titer ≥4.

^a^Sex difference *p*-values derived from multi-level linear (GMT) or logistic regressions (SPR and SCR). Fixed effects included a term for sex and controlled for study year. Random effects included a random intercept on the individual.

### Age, sex, BMI, frailty status, and repeated vaccination are not associated with post-vaccination outcomes

**Fig. 2:**
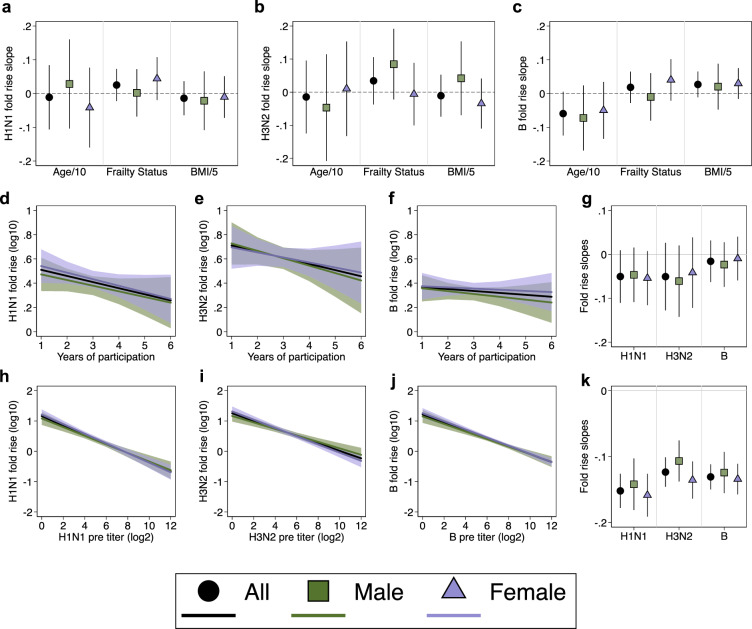
Impact of host factors, repeated vaccination, and pre-vaccination titers on the fold-rise in HAI titers. The relationship of age (in decades, Age/10), frailty status, and BMI (five-unit intervals, BMI/5) with log_10_-transformed fold-rise in titers (post-titer/pre-titer) are shown as slopes for H1N1 (**a**), H3N2 (**b**), and influenza B (**c**). The relationship between increasing years of vaccination and the log_10_-transformed fold-rise in titers are shown for H1N1, H3N2, and influenza B (**d**–**f**), with the slopes summarized (**g**). The relationships between pre-vaccination HAI titers and the log_10_-transformed fold-rise in titers are shown for each vaccine antigen (**h**–**j**), with the slopes summarized (**k**). Estimates and 95% confidence intervals were derived from multi-level mixed-effects models with random intercepts on the individual participant. Models controlled for influenza season and pre-vaccination HAI titers (**a**–**g**), and either controlled for sex (whole population estimates) or used interaction terms between sex and the host factor of interest to derive sex-specific estimates.

We then assessed the relationships between pre-defined host factors (i.e., age, sex, BMI, and frailty status) and post-vaccination strain-specific HAI titer outcomes. When controlling for pre-vaccination titers and influenza season, neither age, frailty, nor BMI individually had statistically significant associations with post-vaccine titers (Supplementary Fig. 1A–C), the fold-rise in titers (Fig. 2a–c), or the odds of seroconversion (S Supplementary Fig. 2A–C) for either H1N1, H3N2, or influenza B. Inclusion of interaction terms in the models allowed for analysis of sex-specific contributions of age, frailty, and BMI as well as sex differences in the effects of these host factors. None of the host factors had a statistically significant association with post-vaccination titers (Supplementary Fig. 1A–C), the fold-rise (Fig. 2a–c), or the odds of seroconversion (Supplementary Fig. 2A–C) for either males or females.

The relationship between the number of years of study participation (measured as a time-varying predictor) and post-vaccination HAI titers outcomes was investigated to evaluate potential negative effects of repeated annual vaccination on the humoral immune response to future vaccines. Vaccination status prior to study enrollment could not be verified, but leveraging the longitudinal design of our study, we sought to quantify whether post-vaccination outcomes declined with additional annual vaccination, and whether this effect differed by sex. There was a non-significant negative trend between the number of years participated and the fold-rise in titers (Fig. 2d–g). These trends were also observed when analyses were repeated using post-vaccination titers (Supplementary Fig. 1D–G) and rates of seroconversion (Supplementary Fig. 2D–G) as outcomes of interest.

### Pre-vaccination HAI titers strongly predict post-vaccination outcomes

Overall, host factors and increasing number of annual vaccinations did not significantly predict any of the post-vaccination antibody titer parameters. Pre-vaccination titers, however, were strong predictors of the fold-rise for H1N1 (slope = −0.15; 95% CI: −0.18; −0.13) (Fig. 2h), H3N2 (slope = −0.12; 95% CI: −0.15; −0.10) (Fig. 2i), and influenza B (slope = −0.13; 95% CI: −0.15; −0.11) (Fig. 2j) (*p* < 0.0001 for testing the null hypotheses that the slope equals zero for each vaccine strain), such that greater pre-vaccination titers were associated with a smaller fold-rise. Analyses were repeated using post-vaccination titers (Supplementary Fig. 1H–K) and odds of seroconversion (Supplementary Fig. 2H–K) as outcomes of interest, and similarly strong associations were observed with the pre-vaccination titers. The strength of these associations suggests that post-vaccination outcomes are primarily determined by pre-existing humoral immunity. Thus, in highly vaccinated populations, such as the older adult participants in this study, pre-vaccination titers are not just confounders to be controlled for in the analysis of post-vaccination humoral immunity but are an outcome of public health importance that illustrate the durability of immunity to influenza from one season to the next. Given the importance of pre-vaccination titers, we focused subsequent analyses on exploring the relationships between host factors and pre-vaccination HAI titers in the context of advanced age and repeated annual vaccination.

### Sex modifies the relationship between age and pre-vaccination HAI titers

**Fig. 3:**
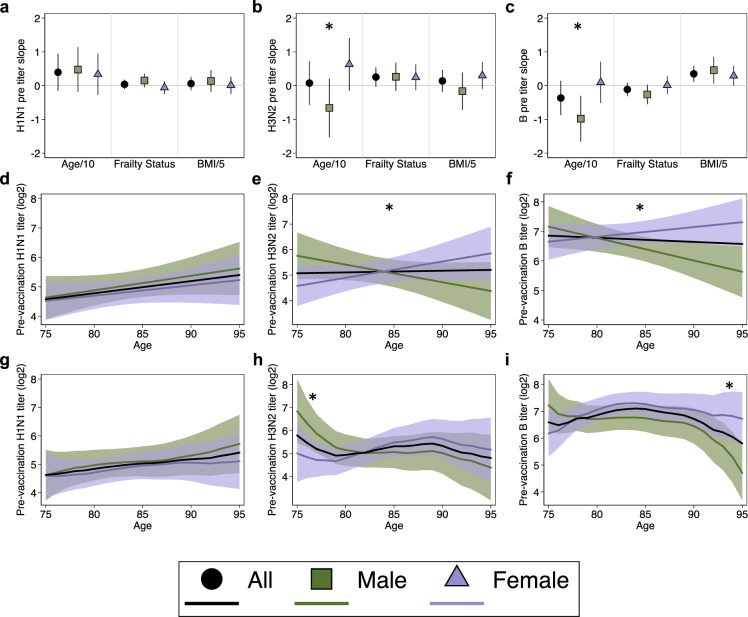
Relationship of age, frailty status, and BMI to pre-vaccination hemagglutination antibody inhibition (HAI) titers. Estimates for the relationship of age in decades (Age/10), frailty status, and BMI (five-unit intervals, BMI/5) to pre-vaccination HAI titers were derived from multilevel mixed-effects models controlling for study year for H1N1 (**a**), H3N2 (**b**), and influenza B (**c**). Expanded age models controlling for frailty and BMI are shown for responses to H1N1 (**d**), H3N2 (**e**), and influenza B (**f**). Expanded models for responses to H1N1 (**g**), H3N2 (**h**), and influenza B (**i**) were then amended to include cubic B-splines for age with knots at 5-year intervals. Models for the whole study population adjusted for sex, while sex-specific estimates included an interaction term allowing effects to differ by sex and are shown with 95% confidence intervals. Asterisks indicate significant sex differences.

Next, we assessed the relationships between age, frailty, BMI, and pre-vaccination titers (Fig. 3a–c). Neither frailty nor BMI were statistically significantly associated with pre-vaccination HAI titers for all participants or in sex-disaggregated subgroups. Further, there were no statistically significant sex differences in the effects of frailty or BMI on pre-vaccination HAI titers against either H1N1, H3N2, or influenza B. A statistically significant sex by age interaction, however, was observed for H3N2 (Fig. 3b) and for influenza B (Fig. 3c), in which HAI titers declined with age among male but not female participants.

To further interrogate the sex-specific effects of aging, expanded models controlling for frailty and BMI, in addition to influenza season, were constructed. Coefficients from the base (i.e., controlling for infleunza season only) and expanded models are shown in Table 3, and results from expanded models are plotted in Fig. 3d–f. For H1N1, HAI titers tended to increase with age for both males and females, but the increase was not statistically significant (males: 0.49 units per decade, *p* = 0.152; females: 0.35 units per decade, *p* = 0.267), nor was the sex difference in slope (*p* = 0.676) (Fig. 3d). For H3N2, while titers again tended to increase with age in females (0.62 units per decade, *p* = 0.121), they tended to decrease with age in males (−0.75 units per decade, *p* = 0.097), leading to a significant sex difference in age slopes (sex by age interaction = 0.137; *p* = 0.010) (Fig. 3e). For influenza B, titers again tended to increase with age in females (0.33 units per decade, *p* = 0.275) but decreased by 0.78 units per decade in males (*p* = 0.023) (Fig. 3f). Like H3N2, the sex difference in the effect of age was significant for influenza B (*p* = 0.005).

**Table 3:** Sex-specific effects of age on pre-vaccination hemagglutination antibody inhibition (HAI) titers.

	Base models^a^						Expanded models^b^					
	Male age effects		Female age effects		Sex difference^c^		Male age effects		Female age effects		Sex difference^c^	
	Change	*p*-value	Change	*p*-value	Difference	*p*-value	Change	*p*-value	Change	*p*-value	Difference	*p*-value
*H1N1*												
Linear	0.047	0.164	0.034	0.286	−0.014	0.674	0.049	0.152	0.035	0.267	−0.014	0.676
Non-linear^d^												
75–80	0.379	0.300	0.146	0.650	−0.023	0.963	0.383	0.301	0.134	0.685	−0.010	0.984
80–85	0.133	0.564	0.233	0.251	−0.256	0.465	0.131	0.574	0.243	0.238	−0.259	0.462
85–90	0.205	0.397	0.109	0.589	−0.156	0.657	0.220	0.377	0.113	0.593	−0.147	0.678
90–95	0.421	0.188	0.066	0.830	−0.253	0.547	0.430	0.186	0.071	0.817	−0.254	0.553
95					−0.609	0.31					−0.612	0.313
*H3N2*												
Linear	−0.067	0.133	0.065	0.103	**0.131**	**0.014**	−0.075	0.097	0.062	0.121	**0.137**	**0.010**
Non-linear^d^												
75–80	−**1.725**	**0.015**	−0.156	0.801	−**1.898**	**0.029**	−**1.776**	**0.012**	−0.297	0.637	−**1.833**	**0.036**
80–85	−0.139	0.711	**0.653**	**0.039**	−0.328	0.469	−0.102	0.786	**0.670**	**0.037**	−0.355	0.435
85–90	0.023	0.953	0.114	0.716	0.463	0.326	−0.048	0.904	0.149	0.651	0.417	0.379
90–95	−0.496	0.39	−0.415	0.430	0.553	0.351	−0.591	0.307	−0.421	0.431	0.614	0.308
95					0.634	0.503					0.784	0.412
*B*												
Linear	−**0.098**	**0.005**	0.009	0.762	**0.108**	**0.007**	−**0.078**	**0.023**	0.033	0.275	**0.111**	**0.005**
Non-linear^d^												
75–80	−0.315	0.516	**0.965**	**0.023**	−1.051	0.087	−0.189	0.693	**1.203**	**0.004**	−1.048	0.080
80–85	−0.106	0.695	0.161	0.486	0.229	0.511	0.038	0.886	0.219	0.330	0.344	0.298
85–90	−0.291	0.306	−**0.534**	**0.019**	0.496	0.165	−0.181	0.515	−0.111	0.633	0.525	0.123
90–95	−**1.799**	**0.000**	−0.434	0.248	0.253	0.572	−**1.649**	**0.000**	−0.233	0.526	0.595	0.168
95					**1.618**	**0.020**					**2.011**	**0.003**

Statistically significant values are bolded.

^a^Base models controlled for study year.

^b^Expanded models controlled for study year, frailty, and BMI.

^c^For linear models, the sex-difference is the age-sex interaction term. For non-linear models, the sex difference is at the beginning of each five-year interval.

^d^Non-linear models include cubic B-splines with knots at 5-year age intervals from 75-95 years.

Both the base and expanded models were then amended to include cubic splines to obtain more granular estimates of the effects of age on HAI titers for males and females. Coefficients for base and expanded non-linear models are shown in Table 3, and results from expanded models are plotted in Fig. 3g–i. The non-linear model for H1N1 did not differ from the linear model, and no significant effects of age within each sex or difference between the sexes were observed (Fig. 3g). Although the trends in the linear models were similar for H3N2 and influenza B, using age as a non-linear predictor revealed that different age categories were driving the overall effects. For H3N2, the increase in pre-vaccination HAI titers with age in females was driven by individuals in the 80–85 age category (increase of 0.67 units; *p* = 0.037), and the decrease in males was driven primarily by people in the 75–80 age category (decrease of 1.78 units; *p* = 0.012) (Fig. 3h). Thus, the sex difference was greatest at the younger end of the cohort (*p* = 0.036 at 75 years of age). Conversely, increasing titers to influenza B with age in females were driven by individuals in the 75–80 age category (increase of 1.2 units; *p* = 0.004), whereas for males there was a sharp decline in HAI titers that occurred in participants 90–95 years of age (decrease of 1.65 units; *p* < 0.0001) (Fig. 3i). Here, the sex difference in pre-vaccination titers was only significant at the oldest end of the cohort (*p* = 0.003 at 95 years of age). Taken together, these data illustrate sex-specific effects of aging on pre-vaccination antibody titers to H3N2 and influenza B, but not H1N1.

To account for the fact that there were several consecutive influenza seasons where the strain included in the vaccine remained constant for either H1N1, H3N2, or influenza B, the above analyses were repeated controlling for viral vaccine stain rather than influenza season. Reanalasis of prevaccination antibody titers using viral vaccine strain rather than influenza season in the models did not change any of the associations between age and sex described above, and the trend of declining pre-vaccination titers in males to H3N2 and influenza B remained (Supplementary Table 7). Further, to illustrate the consequences of ignoring sex as a biological variable, as is commonly encountered in biomedical research^47,48^, analyses were repeated controlling for sex rather than allowing age effects to vary by sex. For H3N2 and influenza B, where the effect of aging was found to be significantly different in males as compared to females, the estimates derived by controlling for sex (black lines in Fig. 3e, f, h, i) were not representative of either males or females. In the linear models, for example, controlling for sex led to the incorrect inference that titers remain constant with age, while the interaction models demonstrate that this is false for both males and females. The goodness-of-fit of models controlling for sex and using an age-by-sex interaction term were compared using Akaike’s Information Criterion (AIC) in Table 4, where lower values indicate better relative goodness-of-fit. For antibody titers to H3N2 and influenza B, despite the penalty for increasing model complexity, fit was improved by including an age-by-sex interaction term that allowed the effect of age to differ by sex. Thus, incorporating sex differences into vaccinology research can lead to more robust analysis.

**Table 4:** Goodness-of-fit comparison of pre-vaccination age models.

	Base models^a^		Expanded models^b^	
*Age-sex interaction*	**–**	**+**	**–**	**+**
*H1N1*				
Linear age	1138.86	1140.69	**1131.05**	1132.87
Non-linear age	1144.47	1151.51	1136.73	1143.72
*H3N2*				
Linear age	1693.38	1689.57	1666.94	**1662.83**
Non-linear age	1694.58	1694.83	1667.28	1667.95
B				
Linear age	1398.31	1393.17	1355.04	1349.28
Non-linear age	1388.09	1378.85	1344.94	**1337.26**

The lowest AIC, corresponding to the best-fit model, is bolded for each virus.

^a^Base models controlled for study year.

^b^Expanded models controlled for study year, frailty, and BMI.

## Discussion

In this multi-season, longitudinal study of older adults over 75 years of age, pre-vaccination titers, rather than host factors or repeated vaccination, strongly predicted all post-vaccination antibody titer outcomes. While it has previously been reported that pre-existing immunity predicts the outcome of vaccination across various age groups^15,17^, the role of sex and age in explaining variability in pre-existing immunity has not been characterized. We report that pre-existing humoral immunity, which reflects the durability of humoral immunity against influenza from previous seasons, displayed an age-by-sex interaction. We found that HAI titers to all three vaccine strains stayed constant in females with age but that HAI titers to H3N2 and influenza B decreased with age in males, leading to significant sex differences in the effect of age for these two vaccine viruses. Thus, sex is a fundamental predictor of the effect of age on pre-existing immunity in this vulnerable population. It has previously been reported that at older ages, there is a male-bias in influenza B infection and hospitalization^49,50^. Our results, therefore, provide a potential mechanism for this sex difference and highlight the need to develop better vaccines or vaccination strategies against influenza for older males.

Because older adults are disproportionally burdened by severe disease and mortality from seasonal influenza, significant effort has been devoted to improving annual vaccination coverage for this vulnerable population. Older adults, particularly those who are over 75 years of age, can thus have decades of repeated annual influenza vaccination. Cumulatively, repeated annual vaccination can lead to high pre-vaccination titers, which we observed in this study and previously reported in younger adult healthcare workers, where mandatory vaccination policies result in exceptionally high rates of immunization^51^. Particularly important in older adults, where formation of *de novo* responses is impaired by immunosenescence^52^, the breadth of pre-existing humoral immunity and the positive predictive value for post-vaccination titers can thus be harnessed to elicit protection^27^. The clinical and scientific implications of this notion are far-reaching and long-term, as the Advisory Committee on Immunization Practices (ACIP) of the CDC has recommended annual influenza vaccination for anyone aged 6 months and older since 2010^53^.

For many vaccines, antibody titers wane over time^54–57^. Influenza vaccines are unique in this respect due to the recommendation for yearly immunization and exceptional antigenic diversity, which alter the dynamics of waning immunity. The constant pre-vaccination HAI titers with age in females seen in our study suggest that females benefit from a booster effect from each successive annual vaccination that appears to prevent antibody waning. This influenza-specific effect has been reported elsewhere, where samples collected from individuals over a 20-year period revealed longitudinal increases in neutralizing titers to influenza^58^. However, our data suggest that this effect is absent in males for H3N2 and influenza B. The reasons for this sex difference are unknown, but may be attributable to the compounding effects of females developing stronger responses to influenza infection and vaccination throughout adulthood^59^, leading to a more robust repertoire of memory B cells that recognize conserved epitopes on drifted virus strains. It is speculated that in older adults, consistently inferior responses among males may manifest as a lack of memory B cells that can be boosted by drifted viruses to counteract waning of antibody over time, thus resulting in decreasing pre-vaccination titers with age.

Notably the sex difference was absent for responses to H1N1 vaccine antigens. A possible explanation for this lies in the differing evolutionary rates of the three vaccine viruses. H1N1 viruses experience slower evolution than H3N2 viruses^60^ and the influenza B/Victoria lineage^61^. In addition, the global co-circulation of B/Yamagata and B/Victoria lineages leads to increased exposure to divergent antigens^62^. Accordingly, over the six influenza vaccine seasons included in our study, and in the past decade, vaccine antigens were significantly more variable for H3N2 and influenza B than for H1N1^63^. It is thus possible that repeated exposure to the same H1N1 strain sufficiently boosted male steady-state immunity to mask sex differences in the immune response. Conversely, for H3N2 and influenza B, sequential exposure to drifted viruses required robust and broad responses to allow for boosting of steady-state immunity, which may have only been present in females. Another possible explanation is immunological imprinting in youth, as it has a lifelong impact on subsequent immune responses to influenza infection and vaccination^23,64^. Individuals in our cohort, born from 1916–1941, may have been exposed to H1N1 in their youth, while the 1918 pandemic virus continued to circulate, but were likely exposed to H3N2 and influenza B later in life^65^. It is, therefore, possible that strong immune imprinting to H1N1 virus strains masked the sex differences otherwise observed for H3N2 and influenza B.

Our study had several strengths and limitations. First, this was an observational study that was not specifically designed to interrogate sex differences in the immune response to influenza vaccination. To overcome small yearly sample sizes, six influenza seasons were pooled together, and statistical methods were used to control for annual variation in vaccine virus strains and repeated measurements on participants. The resulting multi-season nature of this work improves generalizability to future influenza seasons. Secondly, the humoral immune response to vaccination was strain-specific HAI titers, which are the standard in the field, but lack the functional quality of microneutralization assays^66^. Relying solely on serological samples also prohibited mechanistic investigation at the cellular level. In-depth studies of cellular and transcriptional mechanisms underlying the sex differences observed in this cohort are on-going. Third, the lack of racial diversity in our cohort must be noted, as it prohibited us from investigating race as a host factor of interest, which should be considered in future studies. Although the study lacked racial diversity, the cohort was diverse in terms of age at vaccination, allowing us to study effects in the ‘oldest’ old subset. In addition, we were unable to ascertain vaccination history for the participants prior to enrollment in the study. Previous research suggests that influenza vaccination coverage is similar among older men and women^67^, such that it is unlikely that gender differences in vaccination history confounded the results observed. Finally, a major strength of this study is the intersectional approach to analysis, which allowed for interrogation of effects both between and within groups (i.e., between and among males and females), leading to a richer and more nuanced interpretation^68^.

In conclusion, we demonstrate that in highly vaccinated older adults, pre-vaccination HAI titers, rather than age, sex, BMI, frailty, or repeated vaccination, predict post-vaccination parameters of humoral immunity. These pre-vaccination titers change with age in a sex-specific manner, such that older males are particularly vulnerable to lower levels of pre-existing humoral immunity. These findings provide a basis for future studies to investigate the predictive value of host factors and vaccination history in protection from influenza, which could ultimately be a valuable tool in a clinical setting. Further research should focus on elucidating the mechanisms underlying this sex difference, as well as novel vaccination strategies to harness the breadth of pre-existing immunity in older adults to provide better protection against influenza for this vulnerable population.

## Methods

### Study population and protocol

During the 2014–2015 to 2019–2020 influenza seasons, we enrolled community-dwelling older adults above 75 years of age who had not yet received a seasonal influenza vaccine. Individuals who had a history of allergic reaction to influenza vaccines or to eggs, were currently taking oral steroids, or had worsening or new-onset of immune-modulating conditions (e.g., rheumatoid arthritis, hematologic malignancies, etc) were excluded. Study participants came to the Clinical Research Unit at Johns Hopkins Institute of Clinical and Translational Research on the Johns Hopkins Bayview Medical Center campus, or study visits were conducted at participants’ home as needed. A detailed medical history was obtained, vital signs were measured and frailty was assessed as per the Fried Frailty Phenotype^42^. After a pre-vaccination blood draw, participants received HD-IIV3 (Fluzone®High-Dose, Sanofi Pasteur, PA, USA). A second blood sample was collected between 21 and 28 days after vaccine administration (Fig. 1). To focus on the context of repeated annual vaccination, only individuals who participated in a minimum of 4 influenza seasons were included in this analysis.

### Ethics

Written, informed consent was obtained from all participants. The study protocol was approved by the Johns Hopkins School of Medicine Institutional Review Board. The study is registered on clinicaltrials.gov (NCT02200276).

### Hemagglutination inhibition assays

Validated HAI assays were performed by Sanofi Pasteur and used to quantify antibody titers against the three influenza virus strains (H1N1, H3N2, and B) included in each season’s vaccine^69^. Briefly, serum was incubated with type III neuraminidase to eliminate non-specific inhibitors and then with turkey red blood cells to adsorb non-specific agglutinins. Two-fold serial dilutions of sera, beginning at a 1:10 dilution, were then performed in duplicate, and sera were incubated with influenza virus (4 hemagglutination units/25 µl). Turkey red blood cells were then added, and the titer defined as the highest dilution in which hemagglutination of turkey red blood cells was inhibited.

### Definitions and categorization of predictor variables

Sex was used as a dichotomous variable based on self-report. Age was calculated based on the date of vaccination and used as a continuous variable. The frailty assessment was based on the presence or absence of five measurable characteristics: slowed motor performance (by walking speed), poor endurance and energy (by self-report of exhaustion), weakness (by grip strength), shrinking (by unintentional weight loss), and low physical activity (by self-report)^34,42^. Participants with three or more out of these five characteristics were defined as frail, those with one or two as prefrail, and those with none as non-frail. BMI was calculated based on measured height and weight and used as a continuous variable. Influenza season (i.e., 2014–2015 to 2019–2020) was used as categorical variable so as not to imply a linear relationship from year-to-year. The influenza season was included as a dummy variable in analyses to account for possible confounding due to variation in the vaccine composition over time. In sensitivity analyses, viral strain was used as an alternative approach to control for confounding due to antigenic drift. Number of years of study participation was defined as the number of vaccines administered to an individual as part of the study at the time that the outcome was measured each year. Number of years of study participation was used as a time-varying continuous predictor ranging from 1 to 6 (i.e. set to one the first year an individual participated, two the second year an individual participated, etc…).

### Outcome variables

Geometric mean titers were calculated both pre- and post-vaccination. For regression analysis, titers were transformed to a log_2_ scale to achieve an approximately normal distribution. The fold-rise in titer was calculated as post-vaccination titers divided by pre-vaccination titers, and log_10_ transformed to achieve a normal distribution. Seroconversion was defined as achieving a fold-rise ≥4 and used as a binary outcome. Seroprotection was defined as a titer ≥40 and used as a binary outcome.

### Statistical analysis

To account for repeated measures on participants, multi-level mixed-effects models with random intercepts on the individual were used. Although there was missing data in our study, we do not anticipate substantial departure from the missing at random assumption. The mixed-effects method was selected because it is considered to be robust in addressing “non-informative” missing data^70^. Following standard risk factor analysis procedure, the contributions of host factors of interest were first assessed individually. Based on the a priori hypotheses of this analysis, fixed effects of the base models for post-vaccination outcomes adjusted for influenza season, pre-vaccination titers and included interaction terms to allow the effect of the host factor to differ for males and females. Fixed effects of the base models for pre-vaccination titers adjusted for influenza season and included interaction terms to allow effects to differ by sex. Where significant sex differences were found, further analysis controlled for additional covariates, and used cubic B-splines to investigate non-linear relationships^71^. The relative goodness-of-fit of various models were compared using Akaike’s Information Criterion. For graphs, predictions were capped at 95 years of age due to low sample size and large uncertainty in estimates above 95 years. Coefficients were considered statistically significant if 95% confidence intervals did not span the null value of zero (i.e., *p* < 0.05). Analysis was performed in Stata 15 (StataCorp).

### Reporting summary

Further information on research design is available in the Nature Research Reporting Summary linked to this article.

## Supplementary information

Supplementary Information

Reporting Summary

## Data Availability

The data that support the findings of this study are available from the corresponding author upon request.
