# Supplementary material for: Sex-specific effects of aging on humoral immune responses to repeated influenza vaccination in older adults
Source: NPJ Vaccines. 2021 Dec 9;6:147. doi: 10.1038/s41541-021-00412-6 (PMC8660902; doi:10.1038/s41541-021-00412-6)
Supplement: Supplementary file 1 — Supplementary Information [file 41541_2021_412_MOESM1_ESM.pdf]

.

Sex-specific effects of aging on the humoral immune response to repeat vaccination with the high-dose seasonal influenza vaccine in older adults

Supplementary materials

Supplementary tables

1. Pre- and post-vaccination HAI titer outcomes, 2014-2015 season
2. Pre- and post-vaccination HAI titer outcomes, 2015-2016 season
3. Pre- and post-vaccination HAI titer outcomes, 2016-2017 season
4. Pre- and post-vaccination HAI titer outcomes, 2017-2018 season
5. Pre- and post-vaccination HAI titer outcomes, 2018-2019 season
6. Pre- and post-vaccination HAI titer outcomes, 2019-2020 season
7. Sex-specific effects of age on pre-vaccination HAI titers, controlling for viral strain

Supplementary figures

1. Impact of host factors, repeat vaccination and pre-vaccination titers on post-vaccination HAI titers
2. Impact of host factors, repeat vaccination and pre-vaccination titers on the odds of seroconversion

.

Supplementary Table 1. Pre- and post-vaccination HAI titer outcomes, 2014-2015 season

**2014-2015 season**	**All**	**Males**	**Females**	**Sex difference[1]**
**Vaccinations - n**	45	19	26	
**H1N1**				
Pre-vaccination - GMT (95% CI)	83.3 (57.5 - 120.7)	93.5 (60.7 - 143.8)	76.6 (42.7 - 137.3)	0.5987
Post-vaccination - GMT (95% CI)	301.5 (223.3 - 407.0)	322.2 (213.3 - 486.7)	287.2 (183.7 - 448.9)	0.7074
Pre-vaccination SPR - n (%)	33 (73.3)	16 (84.2)	17 (65.4)	0.1676
Post-vaccination SPR - n (%)	44 (97.8)	19 (100.0)	25 (96.2)	.
Fold-rise (log10) - mean (95% CI)	0.559 (0.416 - 0.702)	0.537 (0.382 - 0.693)	0.574 (0.344 - 0.804)	0.8024
Seroconversion rate - n (%)	23 (51.1)	10 (52.6)	13 (50.0)	0.8615
**H3N2**				
Pre-vaccination - GMT (95% CI)	159.0 (106.2 - 238.0)	178.0 (87.0 - 364.3)	146.4 (88.4 - 242.7)	0.6354
Post-vaccination - GMT (95% CI)	485.2 (320.1 - 735.4)	672.1 (326.4 - 1383.6)	382.4 (228.7 - 639.2)	0.1801
Pre-vaccination SPR - n (%)	40 (88.9)	17 (89.5)	23 (88.5)	0.9150
Post-vaccination SPR - n (%)	43 (95.6)	18 (94.7)	25 (96.2)	0.8205
Fold-rise (log10) - mean (95% CI)	0.484 (0.363 - 0.606)	0.577 (0.334 - 0.820)	0.417 (0.292 - 0.541)	0.1937
Seroconversion rate - n (%)	15 (33.3)	8 (42.1)	7 (26.9)	0.2890
**B**				
Pre-vaccination - GMT (95% CI)	458.4 (315.5 - 666.1)	454.0 (298.9 - 689.5)	461.7 (254.4 - 838.3)	0.9644
Post-vaccination - GMT (95% CI)	1153.9 (868.5 - 1533.0)	1296.0 (909.7 - 1846.2)	1060.0 (685.1 - 1640.0)	0.4875
Pre-vaccination SPR - n (%)	44 (97.8)	19 (100.0)	25 (96.2)	
Post-vaccination SPR - n (%)	45 (100.0)	19 (100.0)	26 (100.0)	
Fold-rise (log10) - mean (95% CI)	0.401 (0.302 - 0.500)	0.456 (0.347 - 0.564)	0.361 (0.204 - 0.518)	0.3464
Seroconversion rate - n (%)	18 (40.0)	10 (52.6)	8 (30.8)	0.1432

[1] Sex difference p-values derived from simple linear (GMT) or logistic regressions (SPR and SCR).

Abbreviations & definitions: GMT: geometric mean titer; SPR: seroprotection rate, the proportion of individuals who achieved a titer ≥1:40; Fold-rise: post-vaccination titer divided by pre-vaccination titer, transformed on the log10 scale. Seroconversion rate: the proportion of individuals who achieved a fold-rise in titer ≥ 4.

.

Supplementary Table 2. Pre- and post-vaccination HAI titer outcomes, 2015-2016 season

**2015-2016 season**	**All**	**Males**	**Females**	**Sex difference[1]**
**Vaccinations - n**	68	28	40	
**H1N1**				
Pre-vaccination - GMT (95% CI)	68.1 (51.4 - 90.1)	80.2 (54.6 - 117.7)	60.7 (40.6 - 90.9)	0.3338
Post-vaccination - GMT (95% CI)	199.5 (157.0 - 253.5)	175.3 (125.3 - 245.1)	218.4 (155.1 - 307.5)	0.3714
Pre-vaccination SPR - n (%)	49 (72.1)	20 (71.4)	29 (72.5)	0.9228
Post-vaccination SPR - n (%)	65 (95.6)	27 (96.4)	38 (95.0)	0.7787
Fold-rise (log10) - mean (95% CI)	0.467 (0.373 - 0.560)	0.340 (0.243 - 0.436)	0.556 (0.415 - 0.696)	**0.0217**
Seroconversion rate - n (%)	24 (35.3)	7 (25.0)	17 (42.5)	0.1411
**H3N2**				
Pre-vaccination - GMT (95% CI)	65.8 (48.4 - 89.4)	77.1 (45.2 - 131.4)	58.8 (40.2 - 86.2)	0.3909
Post-vaccination - GMT (95% CI)	673.7 (486.8 - 932.4)	619.4 (363.1 - 1056.8)	714.6 (466.8 - 1093.9)	0.6691
Pre-vaccination SPR - n (%)	47 (69.1)	20 (71.4)	27 (67.5)	0.7302
Post-vaccination SPR - n (%)	68 (100.0)	28 (100.0)	40 (100.0)	0.7302
Fold-rise (log10) - mean (95% CI)	1.011 (0.866 - 1.155)	0.905 (0.693 - 1.117)	1.084 (0.884 - 1.284)	0.2245
Seroconversion rate - n (%)	53 (77.9)	21 (75.0)	32 (80.0)	0.6252
**B**				
Pre-vaccination - GMT (95% CI)	127.8 (96.6 - 169.0)	158.7 (110.6 - 227.8)	109.8 (72.9 - 165.3)	0.1974
Post-vaccination - GMT (95% CI)	382.6 (302.4 - 484.1)	422.9 (304.8 - 586.7)	356.7 (254.3 - 500.2)	0.4813
Pre-vaccination SPR - n (%)	63 (92.6)	28 (100.0)	35 (87.5)	
Post-vaccination SPR - n (%)	68 (100.0)	28 (100.0)	40 (100.0)	
Fold-rise (log10) - mean (95% CI)	0.476 (0.383 - 0.570)	0.426 (0.319 - 0.532)	0.512 (0.368 - 0.655)	0.3696
Seroconversion rate - n (%)	25 (36.8)	9 (32.1)	16 (40.0)	0.5091

[1] Sex difference p-values derived from simple linear (GMT) or logistic regressions (SPR and SCR).

Abbreviations & definitions: GMT: geometric mean titer; SPR: seroprotection rate, the proportion of individuals who achieved a titer ≥1:40; Fold-rise: post-vaccination titer divided by pre-vaccination titer, transformed on the log10 scale. Seroconversion rate: the proportion of individuals who achieved a fold-rise in titer ≥ 4.

.

Supplementary Table 3. Pre- and post-vaccination HAI titer outcomes, 2016-2017 season

**2016-2017 season**	**All**	**Males**	**Females**	**Sex difference[1]**
**Vaccinations - n**	68	31	37	
**H1N1**				
Pre-vaccination - GMT (95% CI)	90.0 (69.0 - 117.4)	92.4 (62.6 - 136.3)	88.0 (60.2 - 128.9)	0.8588
Post-vaccination - GMT (95% CI)	174.1 (135.4 - 224.0)	157.7 (106.2 - 234.2)	189.2 (134.8 - 265.4)	0.4770
Pre-vaccination SPR - n (%)	59 (86.8)	28 (90.3)	31 (83.8)	0.4326
Post-vaccination SPR - n (%)	64 (94.1)	29 (93.5)	35 (94.6)	0.8553
Fold-rise (log10) - mean (95% CI)	0.287 (0.205 - 0.369)	0.232 (0.113 - 0.351)	0.332 (0.216 - 0.448)	0.2284
Seroconversion rate - n (%)	13 (19.1)	5 (16.1)	8 (21.6)	0.5674
**H3N2**				
Pre-vaccination - GMT (95% CI)	119.6 (80.7 - 177.2)	134.1 (74.0 - 243.1)	108.7 (62.8 - 188.0)	0.5990
Post-vaccination - GMT (95% CI)	413.4 (292.8 - 583.5)	477.0 (268.3 - 847.7)	366.7 (237.6 - 565.8)	0.4522
Pre-vaccination SPR - n (%)	54 (79.4)	25 (80.6)	29 (78.4)	0.8180
Post-vaccination SPR - n (%)	65 (95.6)	30 (96.8)	35 (94.6)	0.6663
Fold-rise (log10) - mean (95% CI)	0.539 (0.421 - 0.656)	0.551 (0.366 - 0.736)	0.528 (0.369 - 0.688)	0.8484
Seroconversion rate - n (%)	31 (45.6)	13 (41.9)	18 (48.6)	0.5802
**B**				
Pre-vaccination - GMT (95% CI)	241.1 (183.5 - 316.7)	213.3 (153.6 - 296.0)	267.1 (173.5 - 411.3)	0.4161
Post-vaccination - GMT (95% CI)	601.8 (467.7 - 774.4)	542.5 (388.2 - 758.1)	656.5 (448.3 - 961.4)	0.4560
Pre-vaccination SPR - n (%)	67 (98.5)	31 (100.0)	36 (97.3)	
Post-vaccination SPR - n (%)	68 (100.0)	31 (100.0)	37 (100.0)	
Fold-rise (log10) - mean (95% CI)	0.397 (0.300 - 0.495)	0.405 (0.269 - 0.542)	0.391 (0.247 - 0.534)	0.8801
Seroconversion rate - n (%)	21 (30.9)	7 (22.6)	14 (37.8)	0.1788

[1] Sex difference p-values derived from simple linear (GMT) or logistic regressions (SPR and SCR).

Abbreviations & definitions: GMT: geometric mean titer; SPR: seroprotection rate, the proportion of individuals who achieved a titer ≥1:40; Fold-rise: post-vaccination titer divided by pre-vaccination titer, transformed on the log10 scale. Seroconversion rate: the proportion of individuals who achieved a fold-rise in titer ≥ 4.

.

Supplementary Table 4. Pre- and post-vaccination HAI titer outcomes, 2017-2018 season

2017-2018 season	All	Males	Females	Sex difference[1]
**Vaccinations - n**	87	38	49	
**H1N1**				
Pre-vaccination - GMT (95% CI)	70.6 (54.8 - 90.9)	72.2 (49.7 - 104.7)	69.4 (48.6 - 99.1)	0.8793
Post-vaccination - GMT (95% CI)	207.4 (164.2 - 261.9)	203.2 (139.9 - 295.3)	210.7 (154.8 - 286.7)	0.8802
Pre-vaccination SPR - n (%)	63 (72.4)	27 (71.1)	36 (73.5)	0.8025
Post-vaccination SPR - n (%)	83 (95.4)	36 (94.7)	47 (95.9)	0.7946
Fold-rise (log10) - mean (95% CI)	0.468 (0.383 - 0.553)	0.450 (0.320 - 0.579)	0.482 (0.365 - 0.600)	0.7073
Seroconversion rate - n (%)	31 (35.6)	11 (28.9)	20 (40.8)	0.2534
**H3N2**				
Pre-vaccination - GMT (95% CI)	141.4 (100.3 - 199.2)	145.5 (82.8 - 255.4)	138.3 (88.8 - 215.4)	0.8858
Post-vaccination - GMT (95% CI)	335.9 (246.8 - 457.2)	319.2 (201.1 - 506.9)	349.4 (227.7 - 536.1)	0.7748
Pre-vaccination SPR - n (%)	69 (79.3)	30 (78.9)	39 (79.6)	0.9413
Post-vaccination SPR - n (%)	82 (94.3)	37 (97.4)	45 (91.8)	0.2963
Fold-rise (log10) - mean (95% CI)	0.376 (0.300 - 0.452)	0.341 (0.236 - 0.447)	0.402 (0.292 - 0.513)	0.4329
Seroconversion rate - n (%)	23 (26.4)	8 (21.1)	15 (30.6)	0.3182
**B**				
Pre-vaccination - GMT (95% CI)	264.8 (212.0 - 330.8)	223.3 (160.6 - 310.5)	302.2 (222.5 - 410.6)	0.1817
Post-vaccination - GMT (95% CI)	468.8 (387.3 - 567.4)	385.0 (290.5 - 510.2)	546.2 (421.3 - 708.1)	0.0706
Pre-vaccination SPR - n (%)	87 (100.0)	38 (100.0)	49 (100.0)	
Post-vaccination SPR - n (%)	87 (100.0)	38 (100.0)	49 (100.0)	
Fold-rise (log10) - mean (95% CI)	0.248 (0.177 - 0.319)	0.236 (0.110 - 0.363)	0.257 (0.173 - 0.341)	0.7783
Seroconversion rate - n (%)	12 (13.8)	5 (13.2)	7 (14.3)	0.88

[1] Sex difference p-values derived from simple linear (GMT) or logistic regressions (SPR and SCR).

Abbreviations & definitions: GMT: geometric mean titer; SPR: seroprotection rate, the proportion of individuals who achieved a titer ≥1:40; Fold-rise: post-vaccination titer divided by pre-vaccination titer, transformed on the log10 scale. Seroconversion rate: the proportion of individuals who achieved a fold-rise in titer ≥ 4.

.

Supplementary Table 5. Pre- and post-vaccination HAI titer outcomes, 2018-2019 season

2018-2019 season	All	Males	Females	Sex difference[1]
**Vaccinations - n**	88	40	48	
**H1N1**				
Pre-vaccination - GMT (95% CI)	97.6 (77.5 - 122.9)	110.3 (80.9 - 150.5)	88.2 (62.7 - 124.0)	0.3377
Post-vaccination - GMT (95% CI)	233.4 (192.2 - 283.3)	239.9 (182.1 - 316.2)	228.1 (172.3 - 301.9)	0.7978
Pre-vaccination SPR - n (%)	72 (81.8)	32 (80.0)	40 (83.3)	0.6868
Post-vaccination SPR - n (%)	87 (98.9)	40 (100.0)	47 (97.9)	.
Fold-rise (log10) - mean (95% CI)	0.379 (0.302 - 0.455)	0.337 (0.221 - 0.454)	0.413 (0.309 - 0.517)	0.3313
Seroconversion rate - n (%)	23 (26.1)	7 (17.5)	16 (33.3)	0.0970
**H3N2**				
Pre-vaccination - GMT (95% CI)	119.4 (86.0 - 165.8)	121.8 (75.1 - 197.7)	117.5 (74.0 - 186.5)	0.9136
Post-vaccination - GMT (95% CI)	319.3 (237.0 - 430.2)	322.0 (194.4 - 533.4)	317.1 (219.7 - 457.5)	0.9594
Pre-vaccination SPR - n (%)	70 (79.5)	34 (85.0)	36 (75.0)	0.2512
Post-vaccination SPR - n (%)	83 (94.3)	36 (90.0)	47 (97.9)	0.1470
Fold-rise (log10) - mean (95% CI)	0.427 (0.333 - 0.521)	0.422 (0.303 - 0.542)	0.431 (0.287 - 0.575)	0.9247
Seroconversion rate - n (%)	33 (37.5)	10 (25.0)	23 (47.9)	**0.0292**
**B**				
Pre-vaccination - GMT (95% CI)	271.0 (210.7 - 348.4)	208.2 (146.4 - 295.9)	337.6 (236.7 - 481.4)	0.0566
Post-vaccination - GMT (95% CI)	576.9 (468.0 - 711.1)	448.9 (328.2 - 613.9)	711.0 (538.9 - 938.0)	**0.0287**
Pre-vaccination SPR - n (%)	86 (97.7)	39 (97.5)	47 (97.9)	0.8962
Post-vaccination SPR - n (%)	88 (100.0)	40 (100.0)	48 (100.0)	
Fold-rise (log10) - mean (95% CI)	0.328 (0.262 - 0.394)	0.334 (0.240 - 0.428)	0.324 (0.228 - 0.419)	0.8795
Seroconversion rate - n (%)	21 (23.9)	10 (25.0)	11 (22.9)	0.8195

[1] Sex difference p-values derived from simple linear (GMT) or logistic regressions (SPR and SCR).

Abbreviations & definitions: GMT: geometric mean titer; SPR: seroprotection rate, the proportion of individuals who achieved a titer ≥1:40; Fold-rise: post-vaccination titer divided by pre-vaccination titer, transformed on the log10 scale. Seroconversion rate: the proportion of individuals who achieved a fold-rise in titer ≥ 4.

.

Supplementary Table 6. Pre- and post-vaccination HAI titer outcomes, 2019-2020 season

2019-2020 season	All	Males	Females	Sex difference[1]
**Vaccinations - n**	77	36	41	
**H1N1**				
Pre-vaccination - GMT (95% CI)	49.3 (39.2 - 62.0)	56.8 (42.1 - 76.6)	43.6 (30.8 - 61.8)	0.2556
Post-vaccination - GMT (95% CI)	115.2 (94.0 - 141.0)	114.8 (87.2 - 151.3)	115.5 (85.1 - 156.6)	0.9792
Pre-vaccination SPR - n (%)	49 (63.6)	26 (72.2)	23 (56.1)	0.1449
Post-vaccination SPR - n (%)	72 (93.5)	33 (91.7)	39 (95.1)	0.5438
Fold-rise (log10) - mean (95% CI)	0.368 (0.287 - 0.450)	0.306 (0.197 - 0.415)	0.423 (0.302 - 0.544)	0.1547
Seroconversion rate - n (%)	20 (26.0)	7 (19.4)	13 (31.7)	0.2245
**H3N2**				
Pre-vaccination - GMT (95% CI)	27.5 (21.1 - 35.8)	24.0 (16.9 - 34.1)	30.9 (20.7 - 46.1)	0.3433
Post-vaccination - GMT (95% CI)	200.2 (142.8 - 280.7)	184.4 (105.7 - 321.7)	215.2 (140.3 - 330.0)	0.6524
Pre-vaccination SPR - n (%)	34 (44.2)	15 (41.7)	19 (46.3)	0.6803
Post-vaccination SPR - n (%)	67 (87.0)	30 (83.3)	37 (90.2)	0.3731
Fold-rise (log10) - mean (95% CI)	0.863 (0.740 - 0.986)	0.886 (0.697 - 1.074)	0.843 (0.673 - 1.012)	0.7301
Seroconversion rate - n (%)	52 (67.5)	24 (66.7)	28 (68.3)	0.8792
**B**				
Pre-vaccination - GMT (95% CI)	370.5 (297.1 - 462.1)	304.6 (221.0 - 419.9)	440.1 (323.7 - 598.4)	0.0981
Post-vaccination - GMT (95% CI)	636.4 (523.4 - 773.9)	522.2 (395.1 - 690.1)	757.2 (576.1 - 995.1)	0.0584
Pre-vaccination SPR - n (%)	77 (100.0)	36 (100.0)	41 (100.0)	
Post-vaccination SPR - n (%)	77 (100.0)	36 (100.0)	41 (100.0)	
Fold-rise (log10) - mean (95% CI)	0.235 (0.178 - 0.292)	0.234 (0.156 - 0.312)	0.236 (0.150 - 0.321)	0.9792
Seroconversion rate - n (%)	8 (10.4)	6 (16.7)	2 (4.9)	0.11

[1] Sex difference p-values derived from simple linear (GMT) or logistic regressions (SPR and SCR).

Abbreviations & definitions: GMT: geometric mean titer; SPR: seroprotection rate, the proportion of individuals who achieved a titer ≥1:40; Fold-rise: post-vaccination titer divided by pre-vaccination titer, transformed on the log10 scale. Seroconversion rate: the proportion of individuals who achieved a fold-rise in titer ≥ 4.

.

Supplementary Table 7. Sex-specific effects of age on pre-vaccination HAI titers, controlling for viral strain

	Base Models[1]						Expanded Models[2]					
	Male age effects		Female age effects		Sex difference[3]		Male age effects		Female age effects		Sex difference[3]	
	Change	p-value	Change	p-value	Difference	p-value	Change	p-value	Change	p-value	Difference	p-value
**H1N1**												
Linear	0.063	0.049	0.048	0.098	-0.015	0.662	0.064	0.048	0.05	0.092	-0.014	0.673
Non-linear[4]												
75-80	0.548	0.138	0.358	0.266	-0.052	0.92	0.536	0.153	0.334	0.313	-0.047	0.929
80-85	0.233	0.305	0.295	0.134	-0.242	0.493	0.232	0.315	0.309	0.124	-0.249	0.484
85-90	0.235	0.325	0.186	0.337	-0.18	0.612	0.254	0.301	0.183	0.367	-0.171	0.632
90-95	0.5	0.119	0.132	0.667	-0.229	0.59	0.510	0.117	0.144	0.643	-0.242	0.577
95					-0.598	0.328					-0.608	0.326
**H3N2**												
Linear	-0.058	0.189	0.071	0.071	**0.129**	**0.015**	-0.06	0.178	0.072	0.071	**0.133**	**0.013**
Non-linear[4]												
75-80	**-1.548**	**0.028**	0.006	0.993	**-1.883**	**0.031**	**-1.574**	**0.026**	-0.122	0.845	**-1.806**	**0.040**
80-85	-0.103	0.784	**0.7**	**0.027**	-0.329	0.468	-0.065	0.864	**0.723**	**0.024**	-0.353	0.437
85-90	0.048	0.902	0.139	0.658	0.474	0.314	-0.020	0.960	0.174	0.599	0.434	0.359
90-95	-0.448	0.438	-0.38	0.472	0.564	0.341	-0.538	0.354	-0.390	0.466	0.628	0.296
95					0.633	0.504					0.776	0.417
**B**												
Linear	**-0.075**	**0.029**	0.03	0.336	**0.104**	**0.010**	**-0.055**	**0.100**	0.051	0.095	**0.106**	**0.007**
Non-linear[4]												
75-80	-0.111	0.820	**1.202**	**0.005**	-1.066	0.087	0.019	0.969	**1.426**	**0.001**	-1.061	0.081
80-85	0.048	0.858	0.267	0.248	0.247	0.479	0.176	0.504	0.295	0.191	0.346	0.297
85-90	-0.273	0.341	**-0.443**	**0.054**	0.465	0.194	-0.211	0.453	-0.036	0.877	0.465	0.174
90-95	**-1.643**	**0.000**	-0.354	0.351	0.296	0.510	**-1.523**	**0.000**	-0.148	0.692	0.640	0.141
95					**1.585**	**0.024**					**2.014**	**0.003**

[1] Base models controlled for viral strain.
[2] Expanded models controlled for viral strain, frailty, and BMI.
[3] For linear models, the sex-difference is the age-sex interaction term. For non-linear models, the sex difference is at the beginning of each five-year interval.
[4] Non-linear models include cubic B-splines with knots at 5-year age intervals from 75-95 years.
Statistically significant values are bolded.

.

Supplementary Figure 1. Impact of host factors, repeat vaccination and pre-vaccination titers on post-vaccination titers

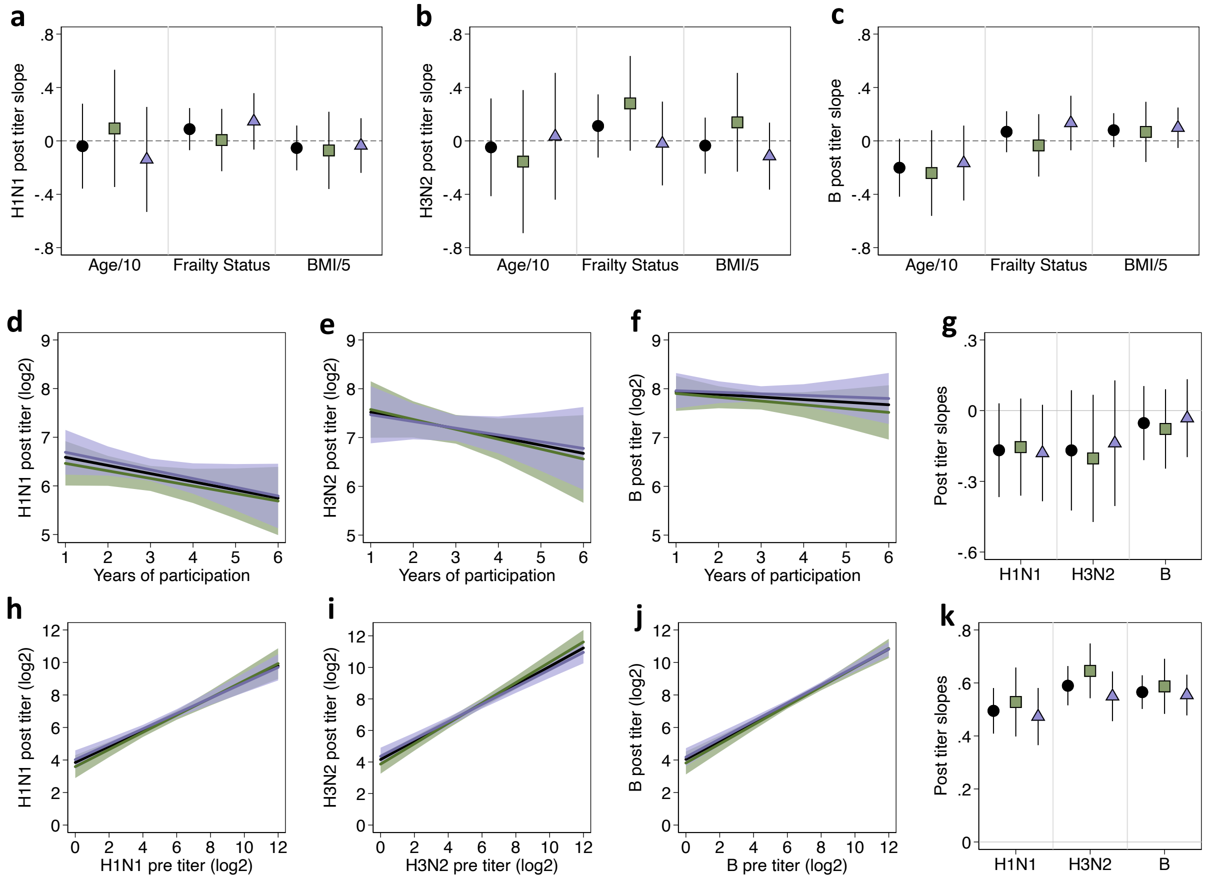

The relationship of age (in decades, Age/10), frailty status, and BMI (five-unit intervals, BMI/5) with log_2-transformed post-vaccination HAI titers are shown as slopes for H1N1 (**a**), H3N2 (**b**) and influenza B (**c**). The relationship between increasing years of vaccination and the log_2-post-vaccination HAI titers are shown for H1N1, H3N2, and influenza B (**d-f**), with the slopes summarized (**g**). The relationships between pre-vaccination HAI titers and the log_2-transformed post-vaccination HAI titers are shown for each vaccine antigen (**h-j**), with the slopes summarized (**k**). Estimates and 95% confidence intervals were derived from multi-level mixed effects models with random intercepts on the individual participant. Models controlled for influenza season and pre-vaccination HAI titers (**a-g**), and either controlled for sex (whole population estimates) or used interaction terms between sex and the host factor of interest to derive sex-specific estimates.

.

Supplementary Figure 2. Impact of host factors, repeat vaccination and pre-vaccination titers on the odds of seroconversion

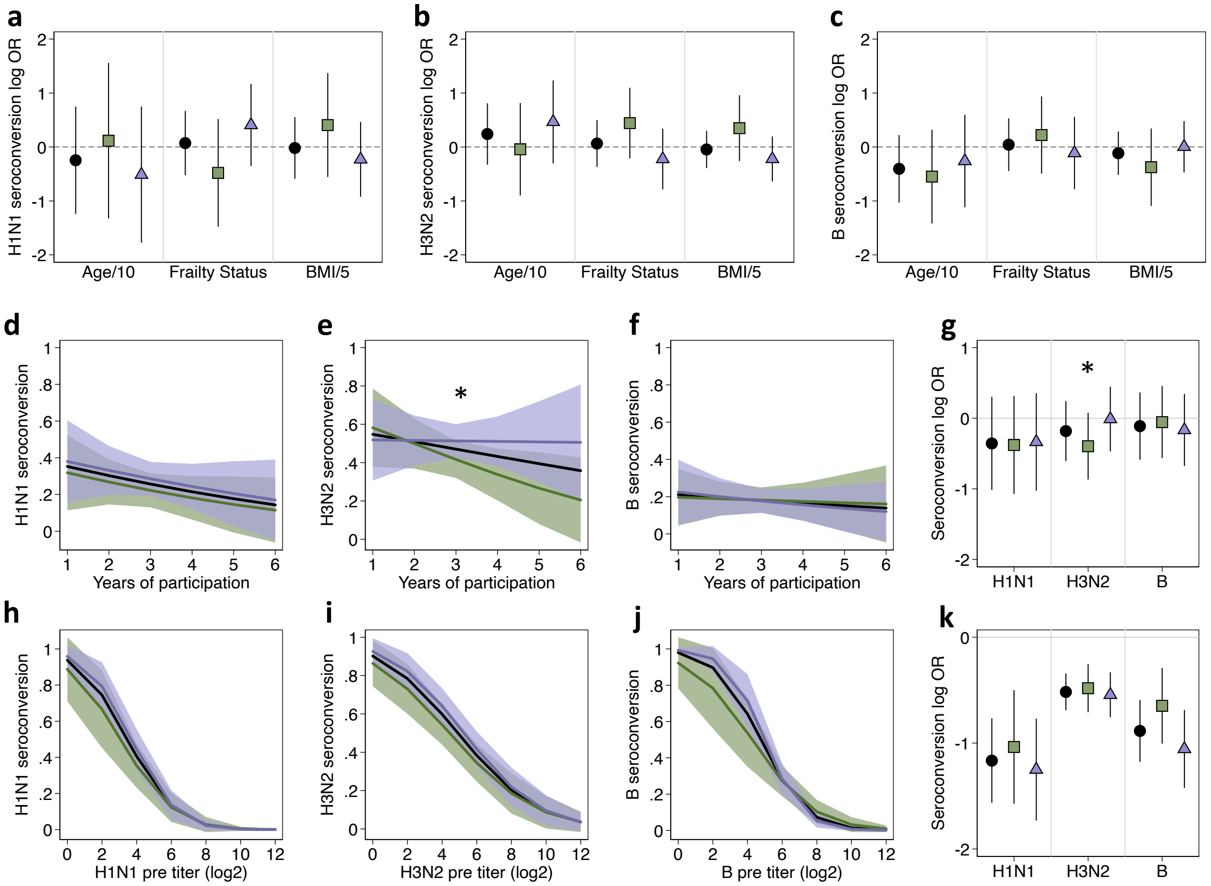

The relationship of age (in decades, Age/10), frailty status, and BMI (five-unit intervals, BMI/5) with log odds of seroconversion are shown H1N1 (**a**), H3N2 (**b**) and influenza B (**c**). The relationship between increasing years of vaccination and the probability of seroconverting are shown for H1N1, H3N2, and influenza B (**d-f**), with the log odds summarized (**g**). The relationships between pre-vaccination HAI titers and the probability of seroconversion are shown for each vaccine antigen (**h-j**), with the log odds summarized (**k**). Estimates and 95% confidence intervals were derived from multi-level mixed effects models with random intercepts on the individual participant. Models controlled for influenza season and pre-vaccination HAI titers (**a-g**), and either controlled for sex (whole population estimates) or used interaction terms between sex and the host factor of interest to derive sex-specific estimates.
