# Supplementary material for: Sex-specific effects of aging on humoral immune responses to repeated influenza vaccination in older adults
Source: NPJ Vaccines. 2021 Dec 9;6:147. doi: 10.1038/s41541-021-00412-6 (PMC8660902; doi:10.1038/s41541-021-00412-6)
Supplement: Supplementary file 2 — Reporting Summary [file 41541_2021_412_MOESM2_ESM.pdf]

nature portfolio

Corresponding author(s): Drs. Sabra L. Klein & Sean X. Leng

Last updated by author(s): Sep 13, 2021

Reporting Summary

Nature Portfolio wishes to improve the reproducibility of the work that we publish. This form provides structure for consistency and transparency in reporting. For further information on Nature Portfolio policies, see our Editorial Policies and the Editorial Policy Checklist.

Statistics

For all statistical analyses, confirm that the following items are present in the figure legend, table legend, main text, or Methods section.

n/a	Confirmed	
☐	☒	The exact sample size (n) for each experimental group/condition, given as a discrete number and unit of measurement
☐	☒	A statement on whether measurements were taken from distinct samples or whether the same sample was measured repeatedly
☐	☒	The statistical test(s) used AND whether they are one- or two-sided<br>*Only common tests should be described solely by name; describe more complex techniques in the Methods section.*
☐	☒	A description of all covariates tested
☐	☒	A description of any assumptions or corrections, such as tests of normality and adjustment for multiple comparisons
☐	☒	A full description of the statistical parameters including central tendency (e.g. means) or other basic estimates (e.g. regression coefficient) AND variation (e.g. standard deviation) or associated estimates of uncertainty (e.g. confidence intervals)
☐	☒	For null hypothesis testing, the test statistic (e.g. F, t, r) with confidence intervals, effect sizes, degrees of freedom and P value noted<br>*Give P values as exact values whenever suitable.*
☒	☐	For Bayesian analysis, information on the choice of priors and Markov chain Monte Carlo settings
☒	☐	For hierarchical and complex designs, identification of the appropriate level for tests and full reporting of outcomes
☐	☒	Estimates of effect sizes (e.g. Cohen's d, Pearson's r), indicating how they were calculated

Our web collection on statistics for biologists contains articles on many of the points above.

Software and code

Policy information about availability of computer code

Data collection	REDCap, version 10.6.1
Data analysis	StataIC 15

For manuscripts utilizing custom algorithms or software that are central to the research but not yet described in published literature, software must be made available to editors and reviewers. We strongly encourage code deposition in a community repository (e.g. GitHub). See the Nature Portfolio guidelines for submitting code & software for further information.

Data

Policy information about availability of data

All manuscripts must include a data availability statement. This statement should provide the following information, where applicable:

- Accession codes, unique identifiers, or web links for publicly available datasets
- A description of any restrictions on data availability
- For clinical datasets or third party data, please ensure that the statement adheres to our policy

The data that support the findings of this study are available from the corresponding author upon request.

Field-specific reporting

Please select the one below that is the best fit for your research. If you are not sure, read the appropriate sections before making your selection.

☒ Life sciences ☐ Behavioural & social sciences ☐ Ecological, evolutionary & environmental sciences

For a reference copy of the document with all sections, see nature.com/documents/nr-reporting-summary-flat.pdf

Life sciences study design

All studies must disclose on these points even when the disclosure is negative.

Sample size	Sample size calculations were originally performed based on the rate of post-vaccination laboratory-confirmed influenza infection in the United States in the 75+ population. The present study is a secondary analysis of the data collected for this primary purpose. For sex-based analysis, sample size was justified by previous work on the impact of biological sex on vaccine-induced immune response. In keeping with our focus on repeated vaccination, sample size was also limited by the number of participants who were vaccinated in at least 4 study seasons.
Data exclusions	To focus on the context of multiple years of repeat vaccination in older adults, individuals who participated in fewer than 4 years were excluded from analysis. This exclusion criteria was set a priori, based on the hypotheses motivating this analysis.
Replication	Laboratory assays were performed in duplicate, and duplicates were assessed for reproducibility. No duplicates varied by more than one titer (i.e. by more than one serial dilution). Where duplicates varied by one titer, the average of the two duplicates was used in analysis. This study also includes 6 influenza seasons (2014-2019), which contributes to reproducibility.
Randomization	Randomization was not relevant to this study as this study was observational and all participants were vaccinated. Covariates of interest included age and sex, which were not possible to randomize.
Blinding	Lab personnel were blinded as to the age and sex of the participants. Blinding as to age and sex was not possible during clinical data collection. No other blinding was necessary as all participants were vaccinated (no control group was included).

Reporting for specific materials, systems and methods

We require information from authors about some types of materials, experimental systems and methods used in many studies. Here, indicate whether each material, system or method listed is relevant to your study. If you are not sure if a list item applies to your research, read the appropriate section before selecting a response.

Materials & experimental systems

n/a	Involved in the study	
☒	☐	Antibodies
☒	☐	Eukaryotic cell lines
☒	☐	Palaeontology and archaeology
☒	☐	Animals and other organisms
☐	☒	Human research participants
☐	☒	Clinical data
☒	☐	Dual use research of concern

Methods

n/a	Involved in the study	
☒	☐	ChIP-seq
☒	☐	Flow cytometry
☒	☐	MRI-based neuroimaging

Human research participants

Policy information about studies involving human research participants

Population characteristics	The study population included 90 individuals over the age of 75 at the time of seasonal influenza vaccination. This included 40 males and 50 females. Each participant contributed 4 to 6 person-seasons to the analysis, such that there were a total 192 person-seasons contributed by males, and 241 contributed by females. Participants were community-dwelling and of all frailty statuses (i.e., robust, pre-frail or frail). Participants reported a variety of comorbid conditions, but those taking immuno-modulatory medications or with comorbid conditions that can affect immune function were excluded.
Recruitment	Participants were mainly recruited from the Johns Hopkins Center on Aging and Health (COAH) Frailty Registry, which included community-dwelling older adults who have expressed interest in participating in clinical research. Additional recruitment was performed through word-of-mouth among participants. This method of recruitment may have led to over-representation of participants who are patients of the Johns Hopkins Medical System, and who may be healthier than the general 75+ population. To address this potential source of bias, study visits were performed at participant's homes as needed, such that individuals with reduced mobility or who were unable to drive were also represented in the study.
Ethics oversight	This study was approved by the Johns Hopkins School of Medicine Institutional Review Board (# NA_00092365)

Note that full information on the approval of the study protocol must also be provided in the manuscript.

Clinical data

Policy information about clinical studies

All manuscripts should comply with the ICMJE guidelines for publication of clinical research and a completed CONSORT checklist must be included with all submissions.

Clinical trial registration	clinicaltrials.gov, NCT02200276
Study protocol	The study protocol can be accessed upon reasonable request from the principle investigator.
Data collection	Data were collected at the Johns Hopkins Asthma & Allergy building, the Johns Hopkins Institute for Clinical and Translational Research Clinical Research Unit, or the participant's home. Data was collected from August 2014 - December 2019.
Outcomes	The primary outcome in this analysis was the strain-specific hemagglutination inhibition titer measured pre- and post-vaccination with the high-dose trivalent seasonal influenza vaccine.
